# The relationship between earthquake risk perceptions, religious orientation, spiritual well-being in individuals with and without earthquake experience: a cross-sectional study

**DOI:** 10.1038/s41598-024-56641-x

**Published:** 2024-03-11

**Authors:** Gönül Gökçay, Ayşe Çevirme, Hülya İncirkuş Küçük, Zeynep Genç Akgün

**Affiliations:** 1 Faculty of Health Sciences, Department of Public Health Nursing, Kafkas University, Central Campus, Kars, Turkey; 2 Faculty of Health Sciences, Department of Public Health Nursing, Sakarya University, Esentepe Campus, Sakarya, Turkey; 3 Health Sciences Institute, Sakarya University, Sakarya, Turkey

**Keywords:** Disaster risk perception, Religious orientation, Spiritual well-being, University student, Psychology, Natural hazards, Risk factors

## Abstract

The physical and psychological effects of earthquakes on individuals with their experience dimension are important. This study aimed to examine the relationship between earthquake risk perception, religious orientation, and spiritual well-being among individuals with and without earthquake experience. The data collection instruments included a socio-demographic information questionnaire, earthquake risk perception scale, religious orientation scale, and three-factor spiritual well-being scale. Statistical evaluations were performed using independent samples *t* test, one-way ANOVA test, Mann–Whitney *U* test, Kruskal–Wallis test (Levene), Pearson correlation, and multiple linear regression analyses. About 59.9% of the participants had experienced an earthquake. Individuals with earthquake experience scored 33.04 ± 7.80 on the earthquake risk perception scale, 100.65 ± 20.80 on the religious orientation scale, and 119.66 ± 18.87 on the three-factor spiritual well-being scale. Those without earthquake experience scored 31.57 ± 7.74, 96.70 ± 18.46, and 114.09 ± 18.04 on the respective scales. The average scores on the earthquake risk perception scale were found to be statistically significant with respect to gender, while the average scores on the religious orientation scale and the three-factor spiritual well-being scale were found to be statistically significant with respect to both gender and substance use. The regression analysis revealed that religious orientation and three-factor spiritual well-being significantly predicted 13.5% of the variance in earthquake risk perception. Studies to increase individuals’ risk perception are important in minimizing the destructive effects of earthquakes in countries in the earthquake zone.

Natural events such as disasters pose a threat to the future of countries and societies by causing material and spiritual harm, resulting in mass casualties and damaging resources^1^. Events like disasters, which make humans feel the limits of their will and power, lead to seeking refuge in Allah, who possesses infinite will and power, and consequently, religious orientation becomes an important way of overcoming problems and coping with them^2–4^

Throughout history and human existence, individuals have faced many problems that affect themselves and their surroundings, and they have tried to cope with economic, social, cultural, and psychological challenges that affect their lives. Among their self-needs, religious orientation holds an important place in bringing individuals to well-being by providing control, reducing uncertainty, and giving meaning and belongingness^5^.

Although our country's territory constitutes approximately 0.5% of the world's territory, 77% of the earthquakes that have occurred since 1900 have occurred at intervals^6^). Earthquakes can cause psychological distress in those who experience or witness them. There are studies in the literature to understand disaster risk perception and religious coping mechanisms^4,7,8^. Research has shown that individuals use religious coping methods to overcome the difficulties after earthquakes such as the Dinar, Gölcük, and Van earthquakes^2^. In another study, the effect of religious beliefs and rituals on overcoming and coping with traumas caused by earthquakes was questioned^8^. The majority of individuals indicated that religious beliefs and rituals were effective in the process of overcoming the trauma. Moreover, individuals defined the cause of the disaster as punishment, warning, test, God's decree, or a natural event, while attributing religious beliefs as a factor that influences the process of coping with difficulties and its outcomes. Believing that the disaster came from God’s reduced anger and increased their ability to be patient. Additionally, individuals emphasized that post-earthquake community support and solidarity increased, and being together and in a crowd helped in making sense of the suffering^9^. In another study, it was observed that individuals' spiritual orientations had a positive contribution to displaying a positive attitude towards life and individual well-being^7,10^.

How individuals perceive the risk of disasters affects the measures, policies, and consequently the damages and consequences of the disaster on society. Studies have approached individuals' risk perceptions based on the type of disaster, the probability and frequency of its occurrence, the number of deaths and injuries, and the economic damage. It has been found that the more severe the experience of the disaster, the stronger the perception of disaster risk^1,11–13^.

Adequate and effective disaster preparedness is essential in every sector to reduce the impact of disasters and enhance disaster resilience to achieve sustainable development goals by 2030. University individuals can play a significant role in promoting and strengthening disaster management activities in society by sharing their knowledge and focusing on disaster preparedness. However, there are very few studies focusing on university individuals' disaster preparedness^14^. In general, individuals' vulnerability to disasters largely depends on their access to finances, housing conditions, knowledge about their socio-environmental situation, and availability. Some researchers still consider university individuals as a low-risk group for disasters because they know how to cope with them. However, the reality is that not all individuals are aware of disasters and may not be well-prepared for them^15^. In the study conducted by Turner, it was noted that students have a low perception of disaster risk, which consequently renders them unprepared for earthquakes^15^. Various studies have examined the disaster preparedness of university students and investigated the interrelationships between socio-economic variables and outcome variables, such as disaster anxiety, disaster experience, perceived preparedness, actual preparedness, disaster knowledge, risk perception^14,16–20^. In Avcı’s study, 92.2% of nursing students stated that they and their environment were not prepared for disasters^21^. In our study, unlike other studies, the effect of religious orientation and spiritual well-being of university students who experienced an earthquake on disaster risk perception was investigated.

While disaster risk perception is effective in understanding and managing the process of individuals' interpretation, taking protective measures, and managing the process, religious orientation holds an important place in overcoming the psychological trauma caused by material and spiritual losses and numerous problems experienced after disasters. After a series of natural disasters such as Hurricane Katrina, Pakistan earthquake, Malaysia flood and Chile earthquake, religious coping mechanisms were used by disaster survivors to cope with their psychological distress and post-traumatic stress disorder. Studies in the literature have generally examined the relationship between spirituality and earthquakes, and findings have been included that religious beliefs often challenge the traumatic effects that may occur on the individual in the period after the earthquake^13,22,23^. In this research, an attempt has been made to determine to what extent the earthquake experience of university students, in relation to their experiences of spiritual well-being, influences their perception of earthquake risk, based on their current situation.

As far as is known in the literature, there is no study examining the religious characteristics and spiritual well-being dimensions of earthquake risk perception. Disaster risk perception; Raising the awareness of university students will increase the momentum in the process of raising social awareness, as it includes the process in which all institutions and organizations are involved in order to reduce the damage, timely and effective response to disasters, and create a healthy environment after the disaster. Furthermore, it is believed that the studies conducted after the recent earthquake disaster in our country will shed light on future research and corrective activities.

## Materıal and method

### Study type

This study is a descriptive research.

### Research questions

What are the levels of earthquake risk perception, religious orientation, and spiritual well-being among individuals with and without earthquake experience? What are the relationship levels between earthquake risk perception, religious orientation, and spiritual well-being among individuals with and without earthquake experience?

### Location and characteristics of the study

This study includes individuals studying at the University's Faculty of Health Sciences and Health Services Vocational School. The units consist of health-related departments such as nursing, midwifery, paramedics, home care, elderly care, medical imaging, medical laboratory, medical documentation, and medical promotion and marketing. The majority of the individuals are undergoing online education due to the recent earthquake in the eastern provinces of the country. Therefore, data were collected through Google Forms in an online environment.

### Population-sample

The study population consists of a total of 3350 individuals studying in both schools. For statistical tests to meet their basic assumptions, the sample size needs to be adequate, and there is a requirement for making good inferences about population parameters (reducing sampling error and providing sufficient power)^24^. In cases where the population is known, it is deemed sufficient to reach 345 individuals with a 95% confidence level and a margin of error of 0.005 using the known population sampling formula (N = 604)^25^. Since data collection is planned to be conducted online in the research, convenience sampling method has been planned for data collection. Convenience sampling method is the easiest, fastest, and most economical way to collect data from the population. It is one of the most commonly preferred methods^26^. By using convenience sampling, individuals were divided into groups and only volunteers participated in the study. The study was completed with the participation of a total of 604 individuals, including 362 individuals with earthquake experience and 242 individuals without earthquake experience. After the study was completed, a post hoc test was conducted using the standardized mean scores of the spiritual well-being scale with GPower 3.1.9.4. software. An effect size of 0.301 was found, and the power analysis conducted with sample sizes of 242 and 362 revealed a power of 0.976.

### Data collection tools

#### Socio-demographic ınformation questionnaire

This form consists of 19 questions developed by the researchers, including socio-demographic characteristics and other influencing factors^27–29^.

#### Earthquake risk perception scale

The scale was developed by Trumbo et al. and adapted into Turkish by Mızrak et al.^29,30^. It consists of 8 items and uses a 5-point Likert scale ranging from 1 (strongly disagree) to 5 (strongly agree). The scale has two subscales: emotional risk perception (items 1–4) and cognitive risk perception (items 5–8). The scale has satisfactory internal consistency with a total Cronbach's alpha of 0.857, emotional risk perception subscale Cronbach's alpha of 0.805, and cognitive risk perception subscale Cronbach's alpha of 0.859. For this study, the overall Cronbach's alpha was found to be 0.932, emotional risk perception subscale Cronbach's alpha was 0.932, and cognitive risk perception subscale Cronbach's alpha was 0.924.

#### Religious orientation scale (ROS)

ROS, developed by Harlak and Eskin was used in this study. It consists of 25 items and three dimensions: Intrinsic Religious Orientation (12 items), Extrinsic Religious Orientation (6 items), and Quest Religious Orientation (7 items)^27^. The internal consistency of the subscales was determined with Cronbach's alpha coefficients, which were found to be α = 0.76 for Intrinsic Religious Orientation, α = 0.70 for Extrinsic Religious Orientation, and α = 0.67 for Quest Religious Orientation. For this study, the overall Cronbach's alpha was 0.928, Cronbach's alpha for the Intrinsic Religious Orientation subscale was 0.862, Cronbach's alpha for the Extrinsic Religious Orientation subscale was 0.771, and Cronbach's alpha for the Quest Religious Orientation subscale was 0.801.

#### Three-factor spiritual well-being scale

The three-factor spiritual well-being scale was developed by Ekşi and Kardaş for adults to determine individuals' understanding and living processes of life in terms of their personal values and meanings, including unique, social, and transcendent aspects^28^. The scale consists of a total of 29 items. The response options in the five-point Likert scale range from 1 (not at all appropriate for me) to 5 (completely appropriate for me). The scale has three subscales: transcendence, harmony with nature, and anomie. The Cronbach’s alpha values of the scale were found to be 0.953 for transcendence, 0.864 for harmony with nature, 0.853 for anomie, and 0.886 for the total scale. The fit indices of the model were as follows: x^2^/sd = 4.11, RMESEA = 0.06, SRMR = 0.50, NFI = 0.90, CFI = 0.92. It was observed that individuals who obtained high scores on the scale had high levels of spiritual well-being, while those who obtained low scores had low levels of spiritual well-being^21^. The scale name has been changed by the authors due to the similarity with a different scale name^31^. When calculating the total score, the items in the anomie subscale (items 3, 7, 11, 15, 19, 23, 26) are reverse scored. For this study, the overall Cronbach's alpha was 0.943, Cronbach's alpha for the transcendence subscale was 0.964, Cronbach's alpha for the harmony with nature subscale was 0.912, and Cronbach's alpha for the anomie subscale was 0.878.

### Data collection method

The survey for the study will be distributed to individuals via social media platforms (WhatsApp, Facebook) using a survey adaptation program (Google Docs). It is estimated that each survey will take approximately 15–20 min to complete.

### Data analysis

The researchers evaluated the data obtained in the study using the SPSS (Statistical Package for Social Sciences) 26.0 software on a computer. Descriptive statistics such as frequencies, percentages, means, and standard deviations were calculated for the data. Skewness and Kurtosis values within the range of ± 2.0 were considered indicative of normal distribution^32^. Values within this range were considered to have a normal distribution. Independent samples *t* tests were used to compare scale means between two groups that exhibited a normal distribution, while one-way ANOVA tests were used for three or more groups that exhibited a normal distribution. For non-normally distributed data, the Mann–Whitney *U* test was used for two groups, and the Kruskal–Wallis test was used for three or more groups. Post-hoc analysis involved conducting Levene's test to assess the homogeneity of variances, and Bonferroni and LSD tests were used for data with homogeneous variances. Pearson correlation analysis was employed to determine the relationships between scales and subscales, logistic and hierarchical regression analysis was used to identify predictors of earthquake risk perception. The significance level for statistical analyses was set at p < 0.05.

### Ethical principles

Permission to use the scales in the study was obtained from the scale developers. Ethical approval was obtained from the Ethics Committee of the Faculty of Health Sciences (Approval No: 81829502.903/32) at the University. Participants in the study were included based on voluntary participation, and no personal identification information was collected. From Sakarya University Faculty of Medicine Non-Invasive Clinical Research Ethics Committee Ethics committee approval was obtained with the number E-71522473–050.01.04-230838-76 This study was performed in line with the principles of the Declaration of Helsinki. Informed consent was obtained from all individual participants included in the study.

### Inclusion criteria for volunteers

Participants had to be individuals of the University's Faculty of Health Sciences and Health Services Vocational School and willing to participate in the study. To be over 18 years old, willing to participate in the study, and not having any physical or cognitive impairments that prevent participation.

### Exclusion criteria for volunteers

Participants who wished to withdraw from the study at any stage were excluded.

### Expected benefits of the study

It is expected that the study will contribute to effective disaster preparedness efforts by determining the relationship between earthquake risk perception, religious orientation, and spiritual well-being in individuals with and without earthquake experience. It aims to provide a foundation for scientific research in public health and make positive contributions to the existing literature.

### Start and end dates of the study and estimated duration

The data collection phase of the study was conducted between March 2023 and April 2023.

## Results

Table 1 presents the descriptive characteristics of individuals with and without earthquake experience. Individuals who had experienced an earthquake accounted for 59.9% of the sample. The mean age of individuals with earthquake experience was 21.38 ± 1.94, while the mean age of individuals without earthquake experience was 21.36 ± 2.47. Among individuals with earthquake experience, 70.2% were female, 30.7% were nursing individuals, 42.3% were first-year individuals, 97.2% were single, 98.1% of those who were married did not have children, 52.8% lived in the city, 75.7% had a moderate income level, 71.3% belonged to a nuclear family structure, and 80.4% did not use any substances. Among individuals without earthquake experience, 78.5% were female, 33.5% were non-paramedic program individuals, 45.5% were first-year individuals, 97.9% were single, 93.5% of those who were married did not have children, 53.7% lived in the city, 80.6% had a moderate income level, 71.1% belonged to a nuclear family structure, and 81% did not use any substances (Table 1).

**Table 1:** Descriptive characteristics of individuals with and without earthquake experience (N = 604).

Variables	Categories	Experienced earthquake (N = 362)	No experienced earthquake (N = 242)		
Age		21.38 ± 1.94	21.36 ± 2.47		

		n	%	n	%
Gender	Female	254	70.2	190	78.5
	Male	108	29.8	52	21.5
University department studied	Nursing	111	30.7	69	28.5
	Midwifery	75	20.7	38	15.7
	Paramedic	75	20.7	54	22.3
	Non-paramedic	101	27.9	81	33.5
Class level	1st year	153	42.3	110	45.5
	2nd year	110	30.4	64	26.4
	3rd year	21	5.8	11	4.5
	4th year	78	21.5	57	23.6
Marital status	Single	352	97.2	237	97.9
	Married	10	2.8	5	2.1
Number of children	No children	158	98.1	87	93.5
	1 child	1	0.6	3	3.2
	3 or more children	2	0.6	3	3.2
Place of residence	City	191	52.8	130	53.7
	Town	101	27.9	67	27.7
	Village	70	19.3	45	18.6
Income level	Low	82	22.7	44	18.2
	Moderate	274	75.7	195	80.6
	High	6	1.7	3	1.2
Family structure	Nuclear family	258	71.3	172	71.1
	Extended family	92	25.4	92	26.9
	Divorced/separated family	12	3.3	12	2.1
Substance use status	No substance use	291	80.4	196	81.0
	Cigarettes	67	18.5	42	17.4
	Alcohol	4	1.1	4	1.7

In Table 2, descriptive data regarding earthquake and spirituality for individuals with and without earthquake experience are presented. It was found that 66% of individuals with earthquake experience lived in earthquake-prone areas, and 85% of them stayed in their homes during earthquakes. Additionally, 68% of them had experienced previous disasters, 53.3% received disaster/earthquake management training, 71.3% had social security for medical examinations, 76.8% had family members employed in income-generating jobs, 79.8% did not receive any spiritual well-being-related education, and 56.1% identified themselves as religious. On the other hand, 78.9% of individuals without earthquake experience lived in earthquake-prone areas, and 80.6% stayed in their homes during earthquakes. Moreover, 93% of them had experienced previous disasters, 62.4% received disaster/earthquake management training, 69.8% had social security for medical examinations, 78.9% had family members employed in income-generating jobs, 81.8% did not receive any spiritual well-being-related education, and 55.8% identified themselves as religious (Table 2).

**Table 2:** Descriptive statistics of earthquake experience and spirituality-related variables among individuals with and without earthquake experience.

Variables	Categories	Experienced earthquake (N = 362)		No experienced earthquake (N = 242)	
		n	%	n	%
Are you living in an earthquake-prone area?	Yes	123	34.0	51	21.1
	No	239	66.0	191	78.9
Where do you stay in the earthquake-prone area?	Tent	12	7.2	7	9.7
	In a tent city	5	3.0	0	0
	In a container	3	1.8	1	1.4
	With relatives/slums	5	3.0	6	8.3
	In my own home	142	85.0	58	80.6
Have you experienced any disaster before?	Yes	246	68.0	17	7.0
	No	116	32.0	225	93.0
Have you received disaster/earthquake management training?	Yes	193	53.3	91	37.6
	No	169	46.7	151	62.4
Do you have social security for medical examinations?	Yes	258	71.3	169	69.8
	No	104	28.7	73	30.2
Do any family members have income-generating employment?	Yes	278	76.8	191	78.9
	No	84	23.2	51	21.1
Have you received training on spiritual well-being?	Yes	73	20.2	44	18.2
	No	289	79.8	198	81.8
Would you consider yourself religious?	Yes	203	56.1	135	55.8
	No	159	43.9	107	44.2

Table 3 presents the earthquake risk perception, religious orientation, and three-factor spiritual well-being scale and subscale averages for individuals with and without earthquake experience. Among individuals with earthquake experience, the earthquake risk perception scale averages were 33.04 ± 7.80 for the overall scale, 14.42 ± 4.64 for emotional risk, and 16.66 ± 3.71 for cognitive risk. The religious orientation scale averages were 100.65 ± 20.80 for the overall scale, 46.22 ± 8.67 for intrinsic orientation, 22.56 ± 4.90 for extrinsic orientation, and 25.97 ± 5.78 for quest orientation. The three-factor spiritual well-being scale averages were 115.13 ± 16.87 for the overall scale, 62.31 ± 12.92 for transcendence, 30.24 ± 5.25 for harmony with nature, and 21.07 ± 6.75 for anomie. Among individuals without earthquake experience, the earthquake risk perception scale averages were 31.57 ± 7.74 for the overall scale, 15.03 ± 4.47 for emotional risk, and 16.43 ± 3.79 for cognitive risk. The religious orientation scale averages were 96.70 ± 18.46 for the overall scale, 46.93 ± 9.02 for intrinsic orientation, 22.79 ± 5.09 for extrinsic orientation, and 26.43 ± 5.62 for quest orientation. The three-factor spiritual well-being scale averages were 114.09 ± 18.04 for the overall scale, 62.14 ± 12.62 for transcendence, 29.71 ± 5.47 for harmony with nature, and 21.95 ± 7.12 for anomie (Table 3).

According to Table 3, earthquake-experienced individuals had significantly higher earthquake risk perception, religious orientation, and three-factor spiritual well-being scores compared to those without earthquake experience (p < 0.05).

**Table 3:** Mean scores of earthquake risk perception, religious orientation, and three-factor spiritual well-being scale and its sub-dimensions among individuals with and without earthquake experience.

Scale and subscales	Number of items	Experienced earthquake	No experienced earthquake	Experienced earthquake	No experienced earthquake	Significance
		Min–max		Mean ± SD		t/p
Earthquake risk perception scale	8	8–40	8–40	33.04 ± 7.80	31.57 ± 7.74	2.267/0.024
Emotional risk perception	4	4–20	4–20	14.42 ± 4.64	15.03 ± 4.47	− 1.620/.106
Cognitive risk perception	4	4–20	4–20	16.66 ± 3.71	16.43 ± 3.79	0.732/.464
Religious orientation scale	25	29–125	25–125	100.65 ± 20.80	96.70 ± 18.46	2.328/0.017
Intrinsic religious orientation	12	15–60	12–60	46.22 ± 8.67	46.93 ± 9.02	− 0.972/0.331
Extrinsic religious orientation	6	6–30	6–30	22.56 ± 4.90	22.79 ± 5.09	− 0.556/0.578
Quest orientation	7	8–35	7–35	25.97 ± 5.78	26.43 ± 5.62	− 1.798/0.073
Three-factor spiritual well-being scale	29	57–143	54–145	119.66 ± 18.87	114.09 ± 18.04	3.695/0.001
Transcendence	15	15–75	15–75	62.31 ± 12.92	62.14 ± 12.62	0.164/0.870
Harmony with nature	7	7–35	7–35	30.24 ± 5.25	29.71 ± 5.47	1.193/0.233
Anomie	7	7–35	7–35	21.07 ± 6.75	21.95 ± 7.12	− 1.539/0.124

Table 4 presents the comparison of earthquake risk perception, religious orientation, and three-factor spiritual well-being scale and subscale averages between individuals with and without earthquake experience in terms of their descriptive characteristics. According to the results, the average scores of the earthquake risk perception scale were found to differ significantly based on gender (t = 4.971, p < 0.001). The average scores of the emotional risk perception subscale showed significant differences based on gender (t = 6.801, p < 0.001) and substance use (KW = 6.441, p = 0.040). Additionally, the average scores of the cognitive risk perception subscale were found to differ based on marital status (U = -2.089, p = 0.037). Furthermore, the average scores of the religious orientation scale showed significant differences based on gender (t = 3.796, p < 0.001) and substance use (KW = 16.552, p < 0.001). The average scores of the intrinsic orientation subscale showed significant differences based on gender (t = 3.705, p < 0.001) and substance use (KW = 12.656, p = 0.002). Similarly, the average scores of the extrinsic orientation subscale differed based on gender (t = 4.266, p < 0.001) and substance use (KW = 10.750, p = 0.005). The average scores of the quest orientation subscale differed based on gender (t = 2.551, p = 0.001), marital status (U = -2.097, p = 0.036), and substance use (KW = 14.991, p = 0.001), (Table 4).

Regarding the three-factor spiritual well-being scale, the average scores differed based on gender (t = 2.510, p = 0.012) and substance use (KW = 8.981, p = 0.011). The transcendence subscale showed differences based on class (F = 2.745, p = 0.042), marital status (U = -2.107, p = 0.035), and substance use (KW = 8.998, p = 0.011). Additionally, the anomie subscale differed based on the field of study (F = 3.964, p = 0.008) (Table 4).

**Table 4:** Comparison of descriptive characteristics and mean scores of earthquake risk perception, religious orientation, and three-factor spiritual well-being scale and its sub-dimensions between individuals with and without earthquake experience.

Değişkenler	Earthquake risk perception scale			Religious orientation scale				Three-factor spiritual well-being scale			
	Emotional risk perception	Cognitive risk perception	Total	Intrinsic religious orientation	Extrinsic religious orientation	Quest orientation	Total	Transcendence	Harmony with nature	Anomie	Total
Gender											
Female	15.40 ± 4.20	16.74 ± 3.67	32.14 ± 7.26	47.29 ± 7.95	23.17 ± 4.62	26.67 ± 5.26	97.13 ± 16.32	62.80 ± 11.69	30.21 ± 4.95	21.25 ± 6.72	115.66 ± 15.81
Male	12.63 ± 4.99	16.09 ± 3.89	28.72 ± 7.95	44.31 ± 10.57	21.24 ± 5.60	25.33 ± 6.68	90.88 ± 21.50	60.72 ± 15.36	29.52 ± 6.30	21.91 ± 7.43	111.78 ± 19.16
Test and p	t = 6.801, p < 0.001	t = 1.865, p = 0.063	t = 4.971, p < 0.001	t = 3.705, p < 0.001	t = 4.266, p < 0.001	t = 2.551, p = 0.011	t = 3.796, p < 0.001	t = 1.763, p = 0.078	t = 1.407, p = 0.160	t = − 1.025, p = 0.306	t = 2.510, p = 0.012
University department studied											
Nursing (a)	14.32 ± 4.63	16.27 ± 3.85	30.59 ± 7.69	46.38 ± 9.32	22.78 ± 5.07	26.27 ± 4.97	95.43 ± 19.14	60.93 ± 13.98	29.55 ± 5.87	20.31 ± 6.38	113.62 ± 18.67
Midwifery (b)	15.22 ± 4.29	16.57 ± 3.63	31.79 ± 7.42	47.42 ± 7.01	23.01 ± 4.24	26.54 ± 6.13	96.96 ± 14.21	63.72 ± 10.52	30.36 ± 4.66	20.94 ± 7.04	116.79 ± 14.98
Paramedic (c)	14.84 ± 4.50	16.47 ± 3.86	31.31 ± 7.69	45.50 ± 9.61	22.33 ± 5.03	26.54 ± 4.58	93.85 ± 19.37	62.29 ± 13.02	29.78 ± 5.37	21.57 ± 6.60	114.78 ± 17.09
Non-paramedic (d)	14.54 ± 4.77	16.92 ± 3.60	31.46 ± 7.56	46.76 ± 8.71	22.54 ± 5.26	26.02 ± 6.02	95.73 ± 18.10	62.60 ± 12.65	30.47 ± 5.17	22.73 ± 7.37	114.18 ± 15.80
Test and p	F = 1.007, p = 0.389	F = 0.947, p = 0.417	F = 0.690, p = 0.558	F = 1.020, p = 0.383	F = 0.442, p = 0.723	F = 0.205, p = 0.893	F = 0.616, p = 0.605	F = 1.184, p = 0.315	F = 1.144, p = 0.331	F = 3.964, p = 0.008*, a > d	F = 0.880, p = 0.451
Class level											
1st year (a)	14.68 ± 4.67	16.57 ± 3.99	31.24 ± 7.96	47.09 ± 8.90	22.97 ± 4.84	26.62 ± 5.47	96.68 ± 17.73	63.92 ± 12.18	30.40 ± 5.27	21.56 ± 7.01	116.59 ± 16.39
2nd year (b)	14.68 ± 4.51	16.68 ± 3.51	31.36 ± 7.23	45.72 ± 8.47	22.12 ± 5.18	25.75 ± 5.83	93.59 ± 17.88	60.78 ± 12.00	29.97 ± 5.13	21.82 ± 6.92	112.81 ± 16.21
3rd year (c)	14.53 ± 4.37	16.09 ± 3.72	30.63 ± 7.61	47.59 ± 6.83	22.88 ± 4.14	27.66 ± 4.43	98.13 ± 13.47	61.69 ± 11.36	29.59 ± 5.33	20.19 ± 6.28	115.09 ± 18.38
4th year (d)	14.65 ± 4.60	16.53 ± 3.55	31.19 ± 7.38	46.11 ± 9.44	22.67 ± 5.11	26.13 ± 6.19	94.92 ± 19.62	61.00 ± 13.71	29.49 ± 5.73	20.96 ± 6.86	113.04 ± 17.76
Test and p	F = 0.010, p = 0.999	F = 0.224, p = 0.880	F = 0.085, p = 0.968	F = 1.102, p = 0.348	F = 1.050, p = 0.370	F = 1.458, p = 0.225	F = 1.302, p = 0.273	F = 2.745, p = 0.042**, a > b, a > d	F = 0.958, p = 0.412	F = 0.770, p = 0.511	F = 2.281, p = 0.078
Marital status											
Single	14.63 ± 4.57	16.52 ± 3.75	31.15 ± 7.60	46.51 ± 8.81	22.64 ± 4.97	26.25 ± 5.71	95.40 ± 18.03	62.13 ± 12.78	30.02 ± 5.33	21.47 ± 6.89	114.49 ± 16.76
Married	16.00 ± 4.84	18.27 ± 2.78	34.27 ± 7.06	46.33 ± 9.32	23.40 ± 5.23	28.60 ± 4.96	98.33 ± 18.60	66.87 ± 12.83	30.40 ± 6.04	19.87 ± 7.91	120.07 ± 19.43
Test and p	U = − 1.247, p = 0.212	U = − 2.089, p = 0.037	U = − 1.648, p = 0.099	U = − 0.034, p = 0.973	U = − 0.722, p = 0.470	U = − 2.097, p = 0.036	U = − 0.980, p = 0.327	U = − 2.107, p = 0.035	U = − 0.667, p = 0.504	U = − 0.851, p = 0.395	U = − 1.744, p = 0.081
Substance use status											
No substance use (a)	14.90 ± 4.52	16.54 ± 3.77	31.44 ± 7.62	47.06 ± 8.23	22.98 ± 4.68	26.70 ± 5.31	96.75 ± 16.72	62.94 ± 12.01	30.04 ± 5.19	21.28 ± 6.85	115.59 ± 16.06
Cigarettes (b)	13.63 ± 4.78	16.79 ± 3.60	30.42 ± 7.45	44.66 ± 10.62	21.47 ± 5.93	25.11 ± 6.74	91.24 ± 21.93	60.04 ± 15.43	30.20 ± 5.84	22.14 ± 7.29	111.35 ± 19.29
Alcohol (c)	14.38 ± 4.10	15.25 ± 4.13	29.63 ± 7.98	37.63 ± 9.08	19.00 ± 4.53	18.88 ± 6.57	75.50 ± 18.47	50.25 ± 11.29	27.13 ± 6.99	21.43 ± 6.91	100.75 ± 17.30
Test and p	KW = 6.441, p = 0.040. a > b	KW = 1.497, p = 0.473	KW = 2.860, p = 0.239	KW = 12.656, p = 0.002. a > b. a > c	KW = 10.750, p = 0.005.a > b	KW = 14.991, p = 0.001. a > b. a > c	KW = 16.552, p < 0.001.a > b	KW = 8.998, p = 0.011.a > c	KW = 3.231, p = 0.199	KW = 0.884, p = 0.643	KW = 8.981, p = 0.011. a > c

*Bonferroni test, **LSD test.

In this study, there were no statistically significant differences in earthquake risk perception, religious orientation, and three-factor spiritual well-being scale and subscale averages between individuals with and without earthquake experience based on their descriptive characteristics such as the number of children, place of residence, income status, and family structure (p > 0.05).

Table 5 presents the correlation analysis between earthquake risk perception, religious orientation, three-factor spiritual well-being scale, and its subscales for individuals with and without earthquake experience. The results indicated a positive and significant relationship between earthquake risk perception scale and intrinsic religious orientation (r = 0.310, p < 0.001), extrinsic religious orientation (r = 0.237, p < 0.001), quest religious orientation (r = 0.208, p < 0.001), religious orientation scale (r = 0.283, p < 0.001), transcendence (r = 0.289, p < 0.001), harmony with nature (r = 0.356, p < 0.001), and anomie (r = 0.199, p < 0.001), as well as the three-factor spiritual well-being scale (r = 0.253, p < 0.001). Moreover, a positive and significant relationship was found between religious orientation scale and transcendence (r = 0.617, p < 0.001), harmony with nature (r = 0.424, p < 0.001), anomie (r = 0.323, p < 0.001), and three-factor spiritual well-being scale (r = 0.492, p < 0.001). Positive and significant relationships were also observed between earthquake risk perception and religious orientation (r = 0.283, p < 0.001), earthquake risk perception and three-factor spiritual well-being (r = 0.253, p < 0.001), as well as religious orientation and three-factor spiritual well-being (r = 0.492, p < 0.001) (Table 5).

**Table 5:** Correlation analysis of earthquake risk perception, religious orientation, three-factor spiritual well-being scale, and its sub-dimensions among individuals with and without earthquake experience.

Korelasyon analizi	Test and p	1	2	3	4	5	6	7	8	9	10	11
1. Emotional risk perception	r	1										
	p	–										
2. Cognitive risk perception	r	0.660	1									
	p	p < 0.001	–									
3. Earthquake risk perception scale	r	0.929	0.891	1								
	p	p < 0.001	p < 0.001	–								
4. Intrinsic religious orientation	r	0.296	0.266	0.310	1							
	p	p < 0.001	p < 0.001	p < 0.001	–							
5. Extrinsic religious orientation	r	0.236	0.192	0.237	0.849	1						
	p	p < 0.001	p < 0.001	p < 0.001	p < 0.001	–						
6. Quest orientation	r	0.222	0.150	0.208	0.765	0.687	1					
	p	p < 0.001	p < 0.001	p < 0.001	p < 0.001	p < 0.001	–					
7. Religious orientation scale	r	0.280	0.230	0.283	0.965	0.908	0.880	1				
	p	p < 0.001	p < 0.001	p < 0.001	p < 0.001	p < 0.001	p < 0.001	–				
8. Transcendence	r	0.244	0.287	0.289	0.587	0.489	0.617	0.617	1			
	p	p < 0.001	p < 0.001	p < 0.001	p < 0.001	p < 0.001	p < 0.001	p < 0.001	–			
9. Harmony with nature	r	0.265	0.397	0.356	0.433	0.367	0.351	0.424	0.760	1		
	p	p < 0.001	p < 0.001	p < 0.001	p < 0.001	p < 0.001	p < 0.001	p < 0.001	p < 0.001	–		
10. Anomie	r	0.200	0.160	0.199	0.336	0.323	0.221	0.323	0.155	0.195	1	
	p	p < 0.001	p < 0.001	p < 0.001	p < 0.001	p < 0.001	p < 0.001	p < 0.001	p < 0.001	p < 0.001	–	
11. Three-factor spiritual well-being scale	r	0.195	0.275	0.253	0.468	0.379	0.502	0.492	0.920	0.789	− 0.158	1
	p	p < 0.001	p < 0.001	p < 0.001	p < 0.001	p < 0.001	p < 0.001	p < 0.001	p < 0.001	p < 0.001	p < 0.001	–

**Figure 1:**
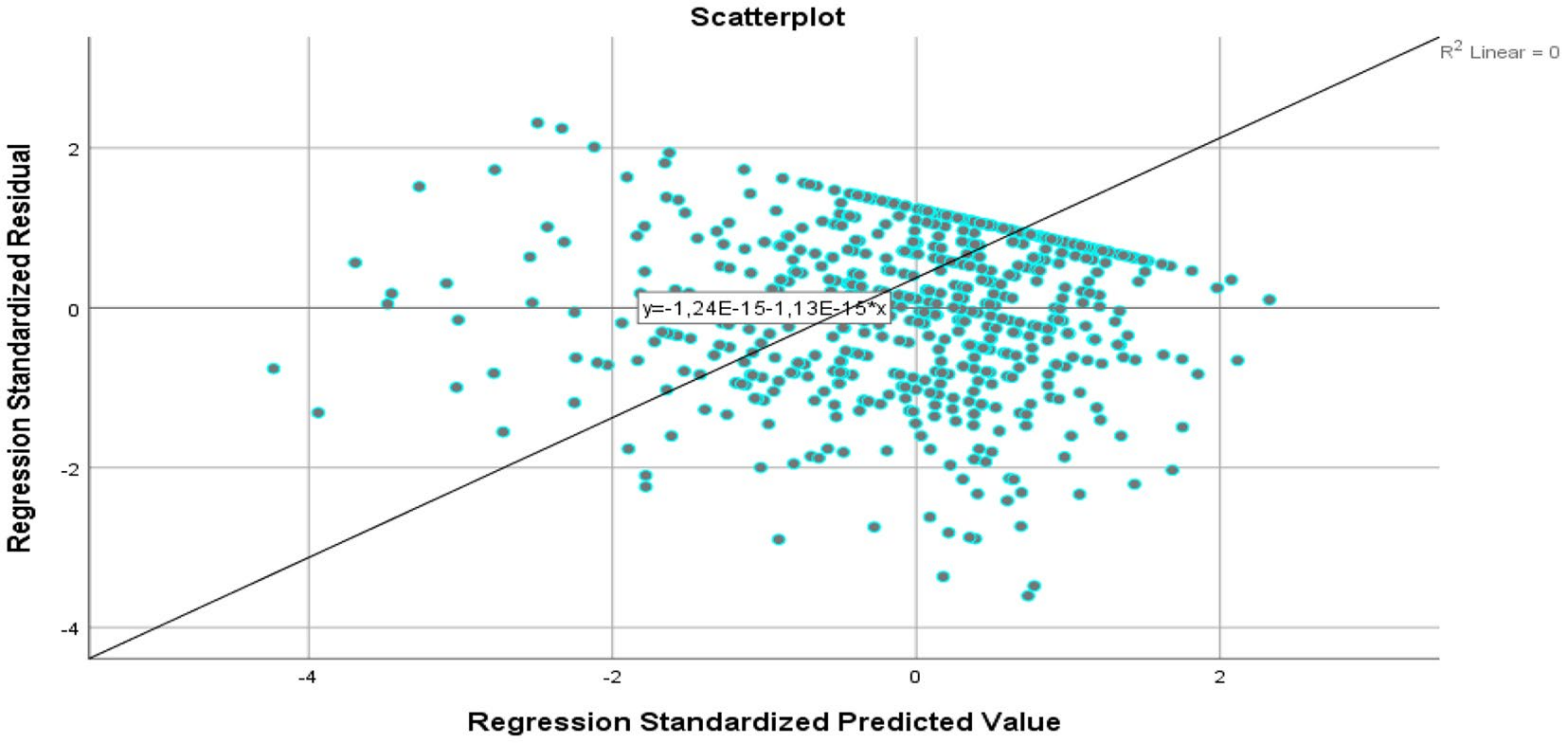
Presentation of factors ınfluencing earthquake risk perception using hierarchical regression analysis.

In Table 6, hierarchical regression analysis of factors influencing individuals' earthquake risk perception is presented. In the first model, it was found that individuals' religious orientation scores significantly predicted earthquake risk perception by 7.8%. In the second model, when the three-dimensional spiritual well-being was added, the statistical significance was not compromised, and the prediction rate increased to 9.4%. In the third model, demographic variables were added, and it was found that being female and receiving education about spiritual well-being, in addition to religious orientation and spiritual well-being, significantly predicted earthquake risk perception by 13.5% (p < 0.001; Fig. 1).

**Table 6:** Presentation of factors ınfluencing earthquake risk perception using hierarchical regression analysis.

Dependent variable: disaster preparedness perception								
Model	Variables	B	Standard error	β	t	p	95.0% confidence	
							Lower bound	Upper bound
1	(Constant)	19.867	1.600		12.417	0.000	16.725	23.009
	Quest orientation	0.119	0.016	0.283	7.228	0.000	0.087	0.151
F(1, 602) = 52.246; R = 0.283; R2 = 0.080; Adjusted R2 = 0.078; Durbin Watson = 2.004; p < 0.001								
2	(Constant)	15.051	2.130		7.066	0.000	10.868	19.235
	Quest orientation	0.088	0.019	0.208	4.679	0.000	0.051	0.125
	Three-factor spiritual well-being scale	0.068	0.020	0.151	3.387	0.001	0.029	0.108
F(2, 601) = 32.313; R = 0.312; R2 = 0.097; Adjusted R2 = 0.094; Durbin Watson = 2.004; p < 0.001								
3	(Constant)	17.635	3.110		5.670	0.000	11.527	23.744
	Quest orientation	0.082	0.019	0.194	4.397	0.000	0.045	0.118
	Three-factor spiritual well-being scale	0.072	0.020	0.159	3.514	0.000	0.032	0.112
	University department studied	0.096	0.268	0.014	0.359	0.720	− 0.430	0.623
	Class level	0.271	0.258	0.042	1.051	0.294	− 0.235	0.776
	Gender	2.815	0.689	0.164	4.085	0.000	1.462	4.169
	Marital status	− 2.904	1.866	− 0.060	− 1.556	0.120	− 6.569	0.761
	Place of residence	− 0.031	0.384	− 0.003	− 0.080	0.936	− .785	0.724
	Income level	− 0.242	0.704	− 0.014	− 0.343	0.732	− 1.625	1.142
	Family structure	− 1.038	0.603	− 0.066	− 1.720	0.086	− 2.223	0.147
	Living in an earthquake-prone area	1.077	0.666	0.064	1.618	0.106	− 0.230	2.384
	Previous experience with disasters	0.913	0.743	0.060	1.230	0.219	− .546	2.373
	Previous experience with earthquake	− 0.772	0.745	− 0.050	− 1.037	0.300	− 2.235	0.691
	Receiving training in disaster management	− 0.149	0.617	− 0.010	− 0.242	0.809	− 1.361	1.063
	Social security status	0.018	0.671	0.001	0.027	0.978	− 1.300	1.336
	Employment status	0.199	0.745	0.011	0.267	0.790	− 1.265	1.662
	Receiving education about spiritual well-being	− 2.812	0.775	− 0.146	− 3.629	0.000	− 4.334	− 1.290
	Self-identification as religious	-0.686	0.607	− 0.045	− 1.130	0.259	− 1.877	0.506
F(17, 586) = 6.553; R = 0.400; R2 = 0.160; Adjusted R2 = 0.135; Durbin Watson = 2.004; **p < 0.001**

As seen in Table 7, the logistic regression model created with earthquake experience status as the dependent variable predicts the earthquake experiences of those who received training in disaster management significantly.

**Table 7:** Prediction results of earthquake experience status using logistic regression.

	B	Standard error	Wald	df	p	95% Confidence	
						Lower	Upper
University department studied	− 0.001	0.163	0.000	1	0.995	0.726	1.374
Class level	− 0.017	0.176	0.009	1	0.925	0.696	1.390
Gender	0.007	0.463	0.000	1	0.989	0.407	2.492
Marital status	0.506	1.122	0.203	1	0.652	0.184	14.966
Place of residence			0.052	2	0.974		
Town	− 0.103	0.510	0.041	1	0.840	0.332	2.449
Village	− 0.035	0.559	0.004	1	0.950	0.323	2.891
Income level	− 0.562	0.458	1.504	1	0.220	0.232	1.399
Family structure	− 0.328	0.375	0.763	1	0.382	0.346	1.503
Living in an earthquake-prone area	0.027	0.428	0.004	1	0.949	0.445	2.375
Previous experience with disasters	− 0.165	0.190	0.759	1	0.384	0.585	1.229
Receiving training in disaster management	3.205	0.485	43.646	1	**0.000**	9.529	63.822
Social security status	0.662	0.401	2.734	1	0.098	0.884	4.251
Employment status	0.331	0.403	0.676	1	0.411	0.633	3.064
Receiving education about spiritual well-being	0.225	0.455	0.243	1	0.622	0.513	3.054
Self-identification as religious	− 0.013	0.502	0.001	1	0.979	0.369	2.638
Religious orientation	− 0.067	0.369	0.033	1	0.855	0.454	1.927
Previous experience with disasters	− 0.007	0.010	0.565	1	0.452	0.974	1.012
Receiving training in disaster management	0.336	1.829	0.034	1	0.854		

## Discussion

The findings of the study examining the relationship between earthquake risk perception, religious orientation, and spiritual well-being of individuals with and without earthquake experience were discussed in line with the literature.

In this study, individuals with earthquake experience were found to have statistically significantly higher scores in earthquake risk perception, religious orientation, and spiritual well-being compared to those without earthquake experience (p < 0.05). Natural disasters are experiences that pose a threat to individuals' physical safety, mental health, and social well-being, with short and long-term adverse effects in these areas. Earthquakes are natural events that endanger human health, disrupt families, and disrupt normal social interactions. It is known that the detrimental effects of earthquakes on mental health can be reduced to the extent that survivors can support themselves spiritually. Religious and spiritual beliefs can serve the purpose of providing individuals with meaning, comfort, and consistency in the face of extreme distress after negative life events^33^. Cui et al. showed in their study that individuals with earthquake experience had higher earthquake risk perception compared to those without earthquake experience^34^. In a study conducted with individuals who witnessed the earthquakes affecting many cities in February 2023, even those trapped under the debris, it was expressed that individuals lacked sufficient knowledge about earthquakes or did not take any precautions to protect themselves. They resorted to prayer and sought refuge in God during and after the earthquake. It was noted that elderly individuals who did not develop any defense mechanisms after witnessing numerous casualties in their immediate surroundings were negatively affected in terms of vulnerability^35^. In another study, university students were asked to generate metaphors regarding the 2023 earthquake. It was found that they produced metaphors such as fear, anxiety, loss, death, and faith^36^. It is believed that experiencing the earthquake, the intensity of the disaster, and the number of injured and deceased individuals in the society influence individuals' perception of earthquake risk.

This study, the average score of the earthquake risk perception scale was found to be significantly higher for women compared to men (p < 0.05). When examining the literature, it is observed, similar to our study, that gender significantly influences disaster risk perception. Women have a higher earthquake risk perception compared to men^37,38^. A study conducted after the hurricane disaster in Florida indicated that gender has an impact on disaster risk perception. Additionally, a study in Taiwan stated that women have a higher earthquake risk perception compared to men^12^. The findings of our study are in line with the existing literature. It has been noted that in women, disaster risk perception is influenced by emotional and environmental factors, which in turn affect awareness and the fulfillment of requirements such as disaster preparedness^39^. The society assigning maternal and spousal roles primarily to women may lead them to have higher levels of responsibility and protective behavior compared to men, which could influence their disaster risk perceptions.

Another factor affecting disaster risk perception is religious orientation. In this study, the average score of the religious orientation scale was found to be significantly higher in women than in men. Positive religious coping methods enhance psychological and physical well-being in individuals and strengthen interpersonal friendships, while negative religiousness can lead to worse physical and mental health, lower quality of life, and depression. In a study by Abdhikari^40^, it was highlighted that individuals who survived a series of natural disasters such as Hurricane Katrina, the Pakistan earthquake, the Malaysia flood, and the Chile earthquake used religious coping mechanisms to deal with psychological distress and post-traumatic stress disorder. The study emphasized the importance of gender differences in using different coping strategies among individuals affected by the earthquake. Female adults who survived tend to use more religious coping and passive coping methods, while surviving males were observed to use active coping, social coping, and self-distracting coping methods more frequently. In a study by Sohrabizade, it was stated that the positive and negative effects of religious orientation after natural disasters affect women more than men, parallel to this study^41^. The higher religious orientation in women compared to men in our study is thought to be due to the traditional acceptance of gender roles in patriarchal societies. Women are traditionally expected to adhere more to private and domestic spheres, while men take on more public roles such as leadership and management. This difference is believed to lead to variations in defense mechanisms developed against challenges between genders.

In this study, the higher religious orientation in women compared to men is thought to be due to the traditional acceptance of gender roles in patriarchal societies, where women are traditionally expected to adhere more to private and domestic spheres, while men take on more public roles such as leadership and management. This difference is believed to lead to variations in defense mechanisms developed against challenges.

In religious beliefs, prayers are considered not to be rejected by God, and adhering to the rules that religion prohibits and commands is accepted^42^. In this study, the average score on the religious orientation scale was found to be significantly higher in non-users compared to substance users. In a study conducted with university students, an inverse relationship between spirituality and alcohol use was reported, indicating lower alcohol and substance use among individuals with higher spirituality. Additionally, some studies have suggested that religious beliefs are effective as alternative treatments in substance use disorder therapy^43–45^. According to Turkish societal norms influenced by religious beliefs, alcohol consumption is among the prohibited behaviors. The reason for the lower religious orientation among alcohol consumers in this study aligns with the existing literature.

In this study, it was found that the average score on the three-factor spiritual well-being scale was significantly higher in women compared to men and in substance users compared to non-users. Spiritual well-being expresses an individual's commitment to oneself, the environment, and the presence of a higher power. It is known that individuals with weak spiritual well-being more frequently experience problems in their mental well-being, feelings of hopelessness, sense of meaninglessness in life, depression, etc.^46^. Similarly, Cherry et al. show through studies conducted after Hurricane Katrina in 2005 and the Deepwater Horizon oil spill in 2010 that spiritual support effectively enhances the resilience of survivors^22^. Timalsina et al. suggest that spirituality can increase individuals' resilience in situations where individuals are vulnerable, such as disasters^23^. Based on all of this, it can be said that individuals' levels of spiritual well-being are important in natural disaster situations.

The hierarchical regression analysis conducted in this study suggests that earthquake risk perception is associated with factors such as religious orientation, spiritual well-being, gender, and spiritual well-being education. In this context, individuals' religious orientations and beliefs can influence their earthquake risk perceptions, and spiritual well-being, defined as finding meaning and purpose in life, can contribute to a strong mental stance in individuals who have experienced disasters. Additionally, the analyses revealed gender differences in earthquake risk perception, with women in society being perceived as more emotional and empathetic. Therefore, it is hypothesized that their perception and reactions to earthquake risk are higher compared to men. Lastly, the analysis focused on the hypothesis that earthquake risk perception may vary among individuals who have received spiritual well-being education. Spirituality, which has a supportive effect on mental health, becomes an alternative in disaster situations where individuals feel more helpless, lonely, and vulnerable. Individuals whoseek support for spiritual well-being find positive responses in their psychosocial experiences. In the face of events, emotions such as anger, fear, resentment, rebellion, stress, and tension are replaced by soothing feelings such as acceptance, tolerance, generosity, and understanding^47–49^. Spiritual well-being education can help individuals explore their inner strengths and increase their mental resilience, enabling them to cope more effectively with stressful situations like earthquakes.

In the literature review conducted regarding the relationship between earthquake risk perception, religious orientation, and spiritual well-being of individuals with and without earthquake experience, no finding was found, which highlights the strength of the study.

Each demographic data was analyzed separately using logistic regression, and a comprehensive model was created with significant variables, revealing a statistically significant relationship between individuals who received disaster management training and their experience with disasters. Despite the absence of prior studies on this topic in the literature, our data underscores the originality of our research.

## Conclusion and recommendations

In this study, the experience of earthquakes has been identified as a significant factor in individuals' disaster risk perception, religious orientations, and spiritual well-being. The study also revealed that gender, religious orientation, well-being, gender, and substance use influence individuals' disaster risk perception. Additionally, individuals' religious orientations and three-dimensional spiritual well-being explain earthquake risk perception by 13.5%.

Experiencing natural disasters such as earthquakes increases individuals' awareness of how they perceive earthquake risks, the damages caused by the disaster to society, and hence their preparedness to protect themselves. In addition, it has been observed that individuals mostly rely on their beliefs and increase their mental resilience through them to overcome the psychological trauma they experience as a result of experiencing a natural disaster such as an earthquake. While it may not be possible for every individual in society to have the experience necessary to protect themselves from earthquakes or other disasters, all individuals carry different risks in terms of various natural disasters. In our country, which is located in an earthquake zone, preventing loss of life, minimizing the environmental and psychological devastation caused by disasters, and enhancing awareness and taking necessary precautions can only be achieved through the education and increased risk perception of all members of society, especially university individuals who are the building blocks of our future.

Considering this situation, strategies can be developed to inform and educate the public correctly and prepare them for disaster situations. In our country, located in the earthquake belt with increasing natural disasters and globalization, preventing loss of life and minimizing environmental and psychological destruction caused by disasters can only be achieved through educating the entire society. Particularly, focusing on educating students, who are the building blocks of our future, and increasing awareness of disaster risk perception is crucial. It is recommended to plan educational activities targeting groups with low risk perception. Moreover, these findings may encourage similar studies to be conducted for other natural disasters.

### Limitations of the study

The inclusion of only university students and healthcare departments, the absence of robust sampling methods such as stratified sampling, the use of non-parametric statistical tests, are limitations of the study. Additionally, data collection through online platforms like Google Forms, and the lack of stratified sampling, are also limitations. This study covers the immediate two-month period following the earthquake. Subsequent studies should also consider the long-term effects of the earthquake. A limitation is that an equal number of students with and without earthquake experience were not included in the study with a larger sample size.

### Recommendations for future researchers

For future studies, it is advised to conduct comprehensive research with a broader and more diverse sample, including multiple universities and countries, to increase awareness and ensure the assumptions of parametric tests are met. Additionally, addressing regional and experiential factors using the stratified sampling method can lead to more reliable results. Furthermore, educational and intervention studies aimed at enhancing disaster preparedness beliefs are suggested. In these interventions, nursing initiatives, taking into account individuals' personal characteristics, religious orientation, and spiritual well-being, are recommended. In addition to the influence of religious beliefs on spiritual well-being, conducting objective research on which rituals or behaviors can positively affect students' coping mechanisms could be instructive for accelerating the return to normalcy processes for individuals affected by earthquakes.

## Competing interests

The authors declare no competing interests.

## Data Availability

The datasets generated during and/or analysed during the current study are available from the corresponding author on reasonable request.
